# Essential Oils for a Sustainable Control of Honeybee Varroosis

**DOI:** 10.3390/vetsci10050308

**Published:** 2023-04-23

**Authors:** Roberto Bava, Fabio Castagna, Ernesto Palma, Mariangela Marrelli, Filomena Conforti, Vincenzo Musolino, Cristina Carresi, Carmine Lupia, Carlotta Ceniti, Bruno Tilocca, Paola Roncada, Domenico Britti, Vincenzo Musella

**Affiliations:** 1 Department of Health Sciences, University of Catanzaro Magna Græcia, CISVetSUA, 88100 Catanzaro, Italy; 2 Interdepartmental Center Veterinary Service for Human and Animal Health, University of Catanzaro Magna Græcia, CISVetSUA, 88100 Catanzaro, Italy; 3 Department of Health Sciences, Institute of Research for Food Safety & Health (IRC-FISH), University of Catanzaro Magna Græcia, 88100 Catanzaro, Italy; 4 Nutramed S.c.a.r.l., Complesso Ninì Barbieri, Roccelletta di Borgia, 88021 Catanzaro, Italy; 5 Department of Pharmacy, Health and Nutritional Sciences, University of Calabria, Rende, 87036 Cosenza, Italy; 6 Pharmaceutical Biology Laboratory, Department of Health Sciences, Institute of Research for Food Safety & Health (IRC-FISH), University of Catanzaro Magna Græcia, 88100 Catanzaro, Italy; 7 Mediterranean Etnobotanical Conservatory, Sersale (CZ), 88054 Catanzaro, Italy; 8 National Etnobotanical Conservatory, Castelluccio Superiore, 85040 Potenza, Italy

**Keywords:** essential oils, *Varroa destructor*, bioinsecticide, contact toxicity, fumigant toxicity, attractant and repellent action, complete exposure, nanoencapsulation, green veterinary pharmacology

## Abstract

**Simple Summary:**

The western honeybee (*Apis mellifera* L.) is one of the most valuable insect species. However, several biological stressors pose a threat to this pollinating insect. Among these, the ectoparasitic mite *Varroa destructor* is currently the most significant concern. In this paper, we offer an updated analysis of the literature on the use of essential oils (EO) to fight against *V. destructor*. Numerous aromatic plants have been subjected to EO extraction to test their varroacidal efficacy in the laboratory or in the field. The results were extremely different even when the same botanical species were used in independent studies. This is undoubtedly related to the enormous variety of methods used to assess the efficacy of acaricides and the variation in plant composition according to origin. This review, in addition to providing an overview of the results, seeks to steer the scientific community towards consistent evaluation methods by pointing out the most valuable research projects currently underway.

**Abstract:**

The *Varroa destructor* parasite is the main obstacle to the survival of honey bee colonies. Pest control mainly involves the use of synthetic drugs which, used with the right criteria and in rotation, are able to ensure that infestation levels are kept below the damage threshold. Although these drugs are easy to use and quick to apply, they have numerous disadvantages. Their prolonged use has led to the emergence of pharmacological resistance in treated parasite populations; furthermore, the active ingredients and/or their metabolites accumulate in the beehive products with the possibility of risk for the end consumer. Moreover, the possibility of subacute and chronic toxicity phenomena for adult honeybees and their larval forms must be considered. In this scenario, eco-friendly products derived from plant species have aroused great interest over the years. In recent decades, several studies have been carried out on the acaricidal efficacy of plant essential oils (EOs). Despite the swarming of laboratory and field studies, however, few EO products have come onto the market. Laboratory studies have often yielded different results even for the same plant species. The reason for this discrepancy lies in the various study techniques employed as well as in the variability of the chemical compositions of plants. The purpose of this review is to take stock of the research on the use of EOs to control the *V. destructor* parasite. It begins with an extensive discussion of the characteristics, properties, and mechanisms of action of EOs, and then examines the laboratory and field tests carried out. Finally, an attempt is made to standardize the results and open up new lines of study in future.

## 1. Introduction

Honeybees play an important ecosystemic role by enabling, through pollination activity, the reproduction of most angiosperm plants [1]. Since its breeding produces goods with significant nutraceutical benefits, this pollinating insect is also highly valued [2]. Unfortunately, we are witnessing the loss of numerous bee colonies worldwide [3,4]. Although the causes of the losses are numerous, the parasite *Varroa destructor* and the infections it carries are one of the main ones [5,6,7,8,9].

*V. destructor* is a honeybee parasitic mite that causes extensive damage to colonies [10]. The life cycle of the parasite comprises the egg, protonymph, deuteronymph, and adult female stages. In the adult stage, the mite parasitises honeybee larvae and adults [11]. In fact, adult mites have buccal portions with well-developed chelicerae (jaws) that are utilized to sting and feed on the fat body of bees [12]. Since the ectoparasitic mite *V. destructor* first appeared in *Apis mellifera*, scientists have developed a wide range of products for parasitosis control. These products fall into one of two categories: hard or soft acaricides [13]. The pyrethroids taufluvalinate, flumethrin, and formamidine amitraz are among the best-known synthetic compounds in the first group. Organic substances including formic acid, oxalic acid, and essential oils (EOs) make up most of the second category [13]. The ability of these products to control *V. destructor* has been extensively studied. Although conventional pest control methods employing synthetic pesticides are appealing due to their ease of use and extreme ability to reduce the impacts of pests, their overuse has endangered the health of honeybees [14,15]. Several acaricides are lipophilic and accumulate in wax [16]. Hive products may contain residual chemicals, and this accumulation may cause long-term exposure to acaricides at levels below the mortality threshold for both adult honey bees and their immature forms [14,15]. It has been shown that even extremely low doses or concentrations can affect the physiology, neurology, metabolism, and/or behavior of honeybees sub-lethally [17]. Due to the sub-lethal consequences, the colony may suffer, as the hive may gradually become less populated [18,19]. Additionally, these acaricides are nowadays less effective due to the development of resistance phenomena.

The need to reduce or replace the synthetic pesticides with natural alternatives has led to the current search for environmentally acceptable treatment methods. The plant kingdom has proven to be quite helpful and is rich in medical resources for the treatment of a variety of human and animal ailments. For this reason, EOs and their monoterpenes are widely studied alternatives in the scientific community for adoption in many pest control programs [20,21,22]. *Ascosphaera apis*, *Paenibacillus larvae*, *Nosema ceranae*, and other honeybee diseases have been successfully treated with these compounds [21,23,24,25]. Compared to hard acaricides, EOs have been highly evaluated and have proven effective as miticides against *V. destructor* over time. The effectiveness of extracts isolated from particular botanical species and the ineffectiveness of others have been noted in numerous publications. The aim of this paper is to review the achievements in the field of EO research. The first section will present an overview of general EO properties, followed by descriptions of extraction procedures, common laboratory investigation techniques, mechanisms of action, and finally an overview of future topics of study.

## 2. Primary and Secondary Metabolites of Plants 

It is common knowledge that the plant kingdom gives us a broad range of natural substances. The two categories of plant constituents are primary and secondary metabolites. The primary metabolites include proteins, amino acids, carbohydrates, and nucleic acids, which are the main macronutrients and are all essential for the development, division, and reproduction of plant cells. Secondary metabolites comprise a more diverse variety of chemical structures than those observed among primary metabolites, and are not involved in the basic metabolisms for plant development, such as photosynthesis and respiration, but in other functions such as defense. The selection of plants for their ability to survive in the environment has led to the diversity of chemical structures that exist today. In their natural habitats, plants are surrounded by numerous potential predators and pathogens. Therefore, plants have evolved protection mechanisms over time that enable them to defend themselves in various ways, as they are sessile organisms. Functional groups included in the molecules of secondary metabolites are fatty acids, hydrocarbons, esters, aldehydes, ketones, alcohols, acetylenic compounds, alkaloids, phenols, and coumarins, to name a few [26]. Secondary metabolic pathways that are directly connected to the main metabolism give rise to secondary metabolites. Shikimic acid and ethyl acetate are the two intermediates that connect the metabolism of glucose with the biosynthetic route of secondary metabolites. In contrast to primary metabolites, secondary metabolites are only distributed in certain parts of plants [27,28]. In particular, they can only be produced and stored by specific organs and glandular tissues (trichomes, glandular cells) and accumulated in vacuoles or extracellular compartments. The most typical ecological functions of secondary metabolites in plants are those that regulate interactions between plants and other species. Secondary metabolites have been shown to have a variety of adaptive properties, including allelopathic qualities (chemical communication and mutual influence between plants) [29], defense against pathogens (phytoalexins) and herbivores, UV protection, and the attraction of pollinators and seed-dispersing animals [30]. Plants have developed direct and indirect protection against herbivores. Direct protection involves the use of silica, secondary metabolites, enzymes, proteins, and organs such as trichomes and thorns that directly affect insect performance. The plant also releases compounds that attract parasites and phytophagous insect predators, which are employed as an indirect form of defense. It has been shown that volatile terpenes and phenylpropanoids from plant species can act as insecticides, food repellents, or supply attractants (for pollination) depending on the insect in question [31]. Secondary metabolites may deter, be indigestible to herbivores, or inhibit oviposition in insects, resulting in population control in young adults [32]. These kinds of metabolites are recognized as active substances because they demonstrate biological activity, and this has attracted interest to a market that seems to be successful in finding new therapeutic applications. These chemicals are thought to number in the hundreds of thousands, and tens of thousands of plant secondary products have been found. Only a small portion of the estimated 308,800 plant species have been investigated, and the vast majority have not been employed to create pesticide-active components. From a wide variety of plant species, scientists have identified about 350 insecticides, more than 800 insect repellents, and a sizable number of insect development inhibitors and regulators; however, it must be considered that few of these have reached the level of commercialization [33]. The main phytochemicals that are currently offered for sale on a global scale include pyrethrins, rotenone, nicotine, ryanodine, sabadilla, and neem products.

## 3. Essential Oils

EOs are a broad term for liquid, highly volatile plant components with a strong, recognizable scent. These are transparent, frequently colorless liquids, soluble in lipids and organic solvents such as alcohol, ether, and fixed oils (they frequently have densities lower than water and typically have high octanol/water partition coefficients). EOs are blends of organic compounds produced by plants as secondary metabolites. They are frequently in charge of giving a particular plant its distinctive aroma. Secretory elements such as glandular trichomes (found in the Lamiaceae family), secretory cavities (found in the Myrtaceae and Rutaceae families), and resin ducts (found in the Asteraceae and Apiaceae families) are linked to the synthesis and accumulation of EOs [34]. EOs have been utilized as medicinal agents for their well-known bactericidal, virucidal, anti-fungal, and anti-parasitic qualities since ancient times. The pharmaceutical, sanitary, cosmetic, and food industries have all seen significant growth in their popularity in recent years. Nevertheless, what makes them particularly intriguing is the part they can play in natural ecosystems, making them an environmentally friendly source of organic insecticides [33]. 

Many plant extracts have historically been asserted to have a variety of toxicological properties against mites, nematodes, and other agricultural pests [35,36]. Recent investigations have shown that some compounds have larvicidal and antifeedant activity, the capacity to postpone development, adult emergence, and ecdysis (moult), as well as the potential to affect mating behavior and, consequently, influence fertility or oviposition [37,38,39,40,41]. Strong-smelling plants that can protect nearby crops include coriander and French marigold. Most insect repellents are made of volatile terpenoids, such as terpinen-4-ol. On the other hand, there are various terpenoids that can operate as attractants. For instance, geraniol will attract honeybees while repelling houseflies. These attractants and repellents have an impact on insect behavior. For pharmacological action, the chemical profile offers a distinctive fingerprint. Many studies have been conducted in recent years to determine the compositional characteristics of the EOs generated from different plant essences. Although there is evidence that minor components also play a significant role, mostly through synergistic effects, it appears that terpenoids and phenolic compounds, which make up a large portion of their composition, are the primary cause of their biological activity [42,43].

In addition to what has already been mentioned, EOs prove to be particularly interesting for another quality. The most alluring attribute of using them as crop protectants is their favorable low mammalian toxicity. For instance, many EOs and their constituents are extensively used as culinary herbs and spices. Such products are routinely exempted by the Environmental Protection Agency from its toxicity data standards. Taking advantage of this situation, certain US companies have recently been able to sell insecticides based on EOs. ValeroTM, a fungicide for grapes, berry crops, citrus fruits, and nuts, and CinnamiteTM, an aphidicide/miticide/fungicide for glasshouse and horticultural crops, are both produced by the firm Mycotech Corporation. Cinnamaldehyde, the active ingredient in both products, is obtained from cinnamon oil [44]. Buzz Away, which contains citronella, cedarwood, eucalyptus, and lemongrass oils, and Green Ban, which contains citronella, cajuput, lavender, safrole from sassafrass, peppermint, and bergaptene from bergamot oil, are two examples of commercial insect repellents [45]. Furthermore, in beekeeping, preparations based on EOs for *V. destructor* parasite control have received approval for marketing. In Italy, for example, Apiguard^®^ products (Vita Europe Ltd., Basingstoke, UK), a patented gel whose special formulation allows the thymol to be released gradually; the vermiculite tablets called ApiLife Var^®^ (Chemicals Laif SPA; Vigonza, Italy) based on EOs of thymol, Eucalyptus Oil, Levomenthol, Camphor; and the product Thymovar^®^ (Andermatt BioVet, Grossdietwil, Switzerland), cellulose sponge strips with thymol, are on sale.

## 4. Composition

Many unique components can be found in EOs. A single oil may include only a few compounds or it may contain a complex mixture of more than one hundred [46]. Terpenes and sesquiterpenes are common components of EOs, as are oxygenated molecules (alcohols, esters, ethers, aldehydes, ketones, lactones, phenols). Usually, there are two or three main components that are concentrated at levels between 20 and 70 percent. For example, carvacrol (30 percent) and thymol (27 percent) are the major components of *Origanum vulgare* EO, linalool (68%) that of *Coriandrum sativum* EO, 1–8 cineole (50%) that of *Cinnamomum camphora* oil, carvone (58%) and limonene (37%) those from the essential seed oil of *Anethum graveolensis*, and finally, menthol (59%) and menthone (19%) are the main components of *Mentha piperita* EO. These secondary metabolites are classified according to the structural bases, biosynthetic routes, or plant types that produce them. The compounds from these groups are frequently conjugated with one or more sugars (the corresponding combined molecules are called glycosides). Typically, the sugars are glucose, galactose or rhamnose. In general, two main groups of different biosynthetic origin can be distinguished [47,48]. The first consists of terpenes and terpenoids, while the second is made up of aromatic and aliphatic components with a low molecular weight. A brief description of the components most commonly found in EOs is given below.

### 4.1. Terpenes and Terpenoids

Terpenes fall into different classes according to their structural and functional nature. Isoprene, a basic chemical compound made up of five carbon atoms, is what this heterogeneous group has in common. The identification of the C5 isoprene unit as a component of the structure of terpenes has been of great help in clarifying their structures. Many terpenes have isoprene units bound in rings, and others (terpenoids) contain oxygen. More precisely, terpenes are not naturally derived from isoprene, which has never been isolated as a natural product of plants, while the true universal precursor of all terpenes is mevalonic acid. The latter is derived from acetyl-CoA and is activated by phosphorylation followed by decarboxylative elimination to give isopentenyl pyrophosphate (IPP), which in turn isomerizes to dimethylallyl pyrophosphate (DMAPP). An enzyme-catalyzed reaction between DMAPP and IPP forms the 10-carbon-atom compound geranyl pyrophosphate, which can easily be hydrolyzed to geraniol, while a further addition of an isopentenyl pyrophosphate molecule generates farnesyl pyrophosphate, the precursor of sesquiterpenes (C15). The distinct terpenes’ functional properties are then ascribed by secondary enzymatic alterations (redox reactions) of the terpene skeleton. According to the number of 5C units, terpenes can be divided into monoterpenes, which are terpenes at 10C (condensation of two isoprene units), sesquiterpenes, which are terpenes at 15C, diterpenes, which are terpenes at 20C, triterpenes, which are terpenes at 30C, and tetraterpenes, which are terpenes at 40C. The majority of the molecules in EOs, or 90 percent of them, are monoterpenes, which are made up of two isoprene units. Terpenoids, which make up more than 40,000 different chemicals, are also included in this large class of secondary metabolites, according to Garcia and Carril (2009) [49]. Unlike terpenes that are characterized by the repetition of isoprene hydrocarbon chains, they can include heteroatoms such as oxygen and a different structural rearrangement. Typical terpenoids such as *Azadirachta indica*, a triterpenoid produced from the neem tree, and pyrethrins from several *Chrysanthemum* spp. are known to have a variety of effects on insect pests, including the suppression of growth and development as well as the prevention of eating and oviposition. [50]. The terpenoides subclasses and other important compounds of EOs are explored below.

### 4.2. Monoterpenes

Monoterpenes consist of two isoprene units; these are the most important molecules, accounting for 90% of EOs. They can be linear (acyclic) or contain rings (cyclic).

Many monoterpenes have been evaluated for their toxicology towards a variety of insects. Particularly, α-pinene, β-pinene, 3-carene, limonene, myrcene, α-terpinene, and camphene have been studied [51]. Epoxypulegone is a monoterpene that, in accordance with Marangoni et al. (2012) [52], inhibits acetylcholinesterase in insects. As a result, insects experience effects such as growth retardation, a reduction in their capacity to reproduce, appetite suppression, and possibly even starvation or direct toxicity.

### 4.3. Diterpenes

The class of compounds called diterpenes has the potential to stop insects from feeding. The insecticidal and antifeedant activities of diterpenoids from the clerodane and neoclerodane families are well known [53]. The efficacy of a number of naturally occurring neoclerodane diterpenoids, generated by *Linaria saxatilis*, and their semi-synthetic derivatives against numerous insect species with different feeding specialisations has been investigated. The antifeedant investigations revealed that the aphid *Myzus persicae* and the oligophagous *Leptinotarsa decemlineata* were the most vulnerable insects. The polyphagous *Spodoptera littoralis* was not suppressed by these diterpenoids, but several of them had post-ingestive antifeedant effects on this insect. In contrast to their toxic or post-ingestive effects, these compounds’ anti-feedant qualities typically varied by species and were more predictable [54].

### 4.4. Triterpenes

A large portion of frequently used insect repellents contain triterpenoids. In this regard, the limonoids from neem (*A. indica*) and chinaberry (*Melia azedarach*) trees, which comprise azadirachtin, toosendanin, and limonin from citrus species, are particularly considered. Anolides, cardenolides, and synthetic saponins are other anti-feedant triterpenoids [55]. The limnoid triterpenes, which are bitter and act as antiherbivore compounds in citrus fruits, are produced by several plants and members of the Rutaceae family. One example is the complex limnoid azadirachtin from *A. indica*, which hinders some insects from feeding and has a number of negative impacts [56].

### 4.5. Sesquiterpene

Sesquiterpenes are another important source of insect repellents. Many insecticidal and antifeedant sesquiterpenes are acknowledged as important inhibitors in interactions between insects and plants [57]. Two feeding inhibitors have been found in the inflorescences of cultivated sunflowers: 3-O-methyl niveusin-A and sesquiterpene lactone angelate argophyllin-A. The sesquiterpene alpha-cyperone, obtained from the tubers of nutgrass (*Cyperus rotundus*), has insecticidal properties against the diamondback moth *Plutella xylostella* [58].

### 4.6. Alkaloids

The broad class of secondary metabolites known as alkaloids is made up of one nitrogen atom that is negatively oxidized. Caffeine, theophylline, theobromine, codeine, thebaine, papaverine, and methylxanthine are some examples of alkaloids [59]. These are nitrogen compounds with modest insecticidal properties that commonly endanger vertebrates [27]. Depending on how their molecules are made, alkaloids can cause negative effects in a variety of ways, but they generally interfere with acetylcholinesterase or sodium channels. Erythrinaline alkaloids highlight their usefulness for crop protection and postharvest storage due to their antifeedant effects. Investigations were also conducted into the stem borer’s limited incidents of attack on maize farms growing under *Erythrina latissima* trees. In post-harvest agricultural procedures, the tree’s seeds and flowers may be employed as a potential bio-pesticide or antifeedant because it is a widespread blooming plant [60]. Two of the most significant natural alkaloids used to manage insect infestations are nicotine and nornicotine. These alkaloids were initially used in the sixteenth century, and by the middle of the nineteenth century, there were 2500 tons in use. Since then, the annual output has fallen and now only covers about 1250 tons of nicotine sulfate and 150 tons of nornicotine, due to their high cost of manufacture, mild odor, acute toxicity to animals, and low insecticidal efficacy.

### 4.7. Phenolic Compounds

Phenolic compounds include a variety of secondary metabolites with defense-related functions, including tannins, lignin, flavonoids, anthocyanins, and furanocoumarins. With more than 8000 phenolic structures recognized and widely distributed across the plant kingdom, phenols, or polyphenols, offer themselves as one of the most varied families of chemicals among secondary metabolites [61]. These phenolic compounds, which are relatively different natural products, all share the existence of at least one aromatic ring with at least one hydrogen modified by a free hydroxyl group or another derivative acting as an ester or heteroside [62]. 

The functional diversity of phenolic compounds is well known; whereas some phenolic compounds provide an attractant function for pollinators or fruit dispersers, other phenolic compounds perform an antagonistic function against herbivores. Moreover, they provide UV protection or fulfill allelochemical functions in neighboring competing plants [63]. One of the key phenolic chemicals that displays insect toxicity is tannin, which binds to salivary proteins and digestive enzymes including trypsin and chymo-trypsin. Because of this, even when insects ingest a lot of tannins, they do not gain weight, get weakened, and may finally die.

One of the biggest and most varied sub-categories of phenolic chemicals are flavonoids. They can be found all over the plant kingdom. The degree of metabolic activity of flavonoids and their metabolites depends on changes in their chemical composition brought on by substitutions such as hydrogenation, hydroxylation, methylation, malonylation, sulfation, and glycosylation. Flavonoids and isoflavones are often found as glycoside derivatives, esters, ethers, or even a combination of these.

## 5. Essential Oils: Extraction Techniques

EOs can be extracted from different plant organs, such as flower, leaf, fruit, bark, seed and even wood and root. However, their extraction yields are usually very low, at around 1%, which may vary depending on plant species and organs [64]. 

EOs can be obtained from raw plant material with different extraction techniques, which can be classified into conventional (or classical) and innovative methods [64,65].

The extraction of the fragrance from plants has been carried out since ancient times, e.g., by Egyptians, Romans and Arabs, and the utilized methods have been improved along the centuries. In the ancient times, EOs were captured into fatty corpses through cold maceration, the so-called “enfleurage” process, or with hot decoction. The improvement of perfumes manufacture was allowed by the development of distillation techniques introduced in the medieval period, thanks to the introduction of the alembic by the alchemist Geber, and to the works of Avicenna, who first distilled ethanol, and also due to the translation of alchemy treaties by the doctors from the School of Salerno in the 12th and 13th centuries. The Eos’ production was then developed on an industrial level in the first half of the 19th century [66].

Hydrodistillation is considered the oldest conventional technique for the extraction of EOs. The plant material is placed into water inside an alembic and they are brought to the boil using a heating source. The utilized apparatus also includes a condenser, which allows one to convert the vapor which comes from the vessel into a liquid, and a decanter is used to collect the condensate and to separate the EO from water. An azeotropic distillation occurs, in which water and EO constituents form a mixture whose boiling temperature is close to but below 100 °C. This allows a co-distillation of the water/EO mixture, which are distilled at the same time [64,65]. Moreover, the hydrodistillation by Clevenger systems allows the recycling of the condensates [64]. However, this method has some drawbacks, such as the presence of artifacts and the alterations of some constituents due to the long contact with boiling water [64]. 

These problems may be overcome using steam distillation, in which there is no direct contact between the plant material and water [64]. Another variant of this kind of extraction is hydro-diffusion, in which the steam is injected into the system from the top to the bottom of the alembic [67].

On the contrary, solvent extraction, in which a hydrocarbon solvent is added to plant material, is not considered among the best techniques, as small amounts of solvent residues may be present in the final product [68]. 

Some particular methods are instead applied to the extraction of EOs from *Citrus* fruits, whose aromatic substances are contained in glands or sacs present in the outer layers of the peel. The volatile compounds localized into the external part of the mesocarp are mechanically removed by cold pressing (also called “expression”), yielding a watery emulsion, followed by recovering the oil using centrifugation [66,69]. Several kinds of cold pressing may be identified. In the manual sponge process, the fruit peel is soaked in water before being pressed between sponges that absorb a mixture of EOs and aqueous components, then separated by decantation. In the “ecuelle” process, the Citrus fruits are instead rolled under pressure in a shallow bowl covered with blunt teeth. Some machines based on the sponge or the “ecuelle” processes (“sfumatrici” and “pellatrici”, respectively), are particularly used in Italy and are utilized at an industrial scale [69]. 

Even if the extraction of EOs in the perfume industry is considered to be cleaner than heavy chemical industries, its environmental impact is greater than it first appears, as the EO extraction requires high quantities of plant material, energy and water as cooling agents [70]. For these reasons, together with the conventional extraction techniques, new advanced methods have been introduced over the past years, such as supercritical fluid extraction, subcritical water extraction, ultrasound-assisted and microwave-assisted extractions [71].

These techniques are considered “green”, as they require shorter times and are able to improve the yields and quality of EOs, allowing at the same time a reduced consumption of energy and solvents [72].

The supercritical fluid extraction (FSE) of EOs is performed using carbon dioxide (CO_2_) as its low polarity makes this molecule suitable for the extraction of volatile compounds. The use of this solvent presents many advantages in EO production. The critical point (72.9 atm and 31.2 °C) can be easily reached and does not induce damage to the thermolabile molecules. Moreover, carbon dioxide is nontoxic and it can be easily eliminated by simple depression without leaving any traces [64,73,74,75,76]. Compared to conventional processes, such as hydrodistillation and steam distillation, this method allows one to obtain high yields with shortened process times [77]. 

In subcritical water extraction (SWE), water is used at high pressures (>20 bar) and at temperatures ranging between 100 and 374 °C (critical temperature) [78]. Under these conditions, the water polarity decreases, and nonpolar components are solubilized and extracted from plant material. This technique is also referred to as pressurized low-polarity water extraction (PLPWE) or pressurized hot water extraction (PHWE) [71].

Ultrasound-assisted extraction (UAE) is used for the isolation of volatile compounds from aromatic plants at room temperature with the use of organic solvents [79]. In this technique, the breakdown of cavitation bubbles generated during ultrasonication generates micro-jets able to destroy the glands containing the EOs constituents and facilitate their release [67,80].

Finally, microwave-assisted extraction (MAE) can be successfully used for the extraction of EOs from aromatic plants [81]. The microwave-assisted distillation (MWHD) is based on the combination of distillation and microwave heating performed at atmospheric pressure. The matrix is placed with water into a reactor which is placed inside a microwave oven. Furthermore, one of the more recent techniques is the solvent-free microwave-assisted extraction (SFMAE), performed without using any organic solvent or water [82,83,84].

UAE and MAE are successfully applied also to the extraction of EOs from *Citrus* spp. [85,86].

## 6. Mechanism of Action

EOs interfere with insects’ metabolic, biochemical, physiological, and behavioral processes. Insects can consume, breathe in, or absorb EOs through their body surface. Therefore, the EO begins to act after it has been absorbed at various levels. Toxic action is mainly expressed at the nervous system level. EOs develop a distinct chemical profile depending on the botanical source and species, and may interfere with acetylcholinesterase, GABA, and octopamine receptor activity. Let us begin to analyze its interference with octopamine. The multifunctional invertebrate chemical octopamine (OA) is comparable in structure and function to the vertebrate hormone noradrenaline. The biogenic amine octopamine serves several different purposes in insects [87]. It has been found to perform three distinct purposes as a neurotransmitter, neurohormone, and neuromodulator [88,89]. It is involved in regulating different facets of insect behavior, including arousal level. Moreover, it is essential for insects’ social behavior, aggression, and stress reaction. Based on pharmacological criteria, OA interacts with at least two types of receptors, referred to as octopamine-1 and octopamine-2, to achieve its effects [90,91]. Intracellular calcium levels increase when OA binds to the first type of receptor, which in turn boosts the levels of cAMP. As an alternative, binding to the second type of receptor results in a direct increase in cAMP levels. There are many components of EOs that have pharmacological effects and have been demonstrated to influence insects’ octopaminergic systems [92]. Increases in cAMP were produced by the compounds eugenol and α-terpineol. Nevertheless, geraniol and citral decreased cAMP levels more significantly. The same EOs reduced the affinity of [3H]-OA for receptors. It is interesting to notice that just cinnamic alcohol increased the OA level of *Blatella germanica* by more than 20 times. In a study by Enan [93], it was discovered that the toxicity of eugenol, cinnamyl alcohol, 2-phenethyl propionate, and trans-anethole is caused by their interactions with the OA receptor. EO compounds such eugenol, trans-anethole, and 2-phenethyl propionate increased Ca^2+^ concentrations in HEK-293 cells that were expressing OAr from *Periplaneta americana* and *Drosophila melanogaster*. Nevertheless, cAMP levels in these cells were decreased by eugenol and increased by trans-anethole. All three of these EO components significantly decreased the binding of [3H]-yohimbine (ligand of OAr). Hollingworth et al. (1984) [94] claimed that insects’ whole neural systems halted when octopamine activity was interfered with.. The lack of octopamine receptors in vertebrates is most likely what accounts for the great selectivity of EOs as insecticides. As a result, an effective biological target for insect control is the octopaminergic system. 

The GABA-gated chloride channels are another route via which EOs act, which may account for the pesticides’ rapid impact against certain pests. [95]. Gamma-aminobutyric acid (GABA) is the main inhibitory neurotransmitter in the musclular and nervous system of both mammals and insects. It binds to specific GABA receptors on synaptic or extrasynaptic membranes. Animals have two different types of GABA receptors: ionotropic (GABAArs) and metabotropic (GABABrs). Studies on EOs’ effects on GABArs, a group of receptors renowned for their ionotropic properties, are widely available. Similarities exist between the ionotropic GABArs seen in insects and vertebrates. GABArs are crucial in mediating the inhibitory effect on neurotransmission in the nervous system of insects, just as in vertebrates. However, insect GABArs are structurally and pharmacologically distinct from mammalian GABArs, making them a particularly fascinating target for the development of new insecticides. The Cl^-^ current induced by the GABA neurotransmitter is amplified by thymol, menthol, and other compounds. Many EO constituents, such as camphor, carvone, menthone, linalool, and α-terpineol, have no effect on the GABAArs Cl^-^ current. The interactions of the EO components with GABA receptors are influenced by their chemical composition. Different EO stereoisomers have different capacities for controlling GABA receptors; (+)-menthol and (+)-borneol are more active than (−)-menthol and (−)-borneol. The presence of a functional group is also crucial. Alcohols such as thymol, menthol, and borneol have a greater modulatory impact on the GABAArs than ketones (linalool, α-terpineol). Several studies have been conducted to pinpoint the GABArs’ binding sites for the EO components. Such experiments are difficult to perform in natural neuronal membranes, however, because EOs are lipophilic compounds that might change cellular membranes in a non-specific way. Studies comparing EOs to other GABAAr ligands provide the majority of data on how EOs interact with GABAArs. Although these investigations can only provide inferential support for the existence of EO component binding sites in the GABAArs, they should be complemented with more conclusive methods. It has been suggested that low-molecular-weight (LMW) terpenoids enter through the tracheae because they may be too lipophilic to dissolve in the haemolymph after passing through the cuticle [96]. Recent research indicates that target sites on receptors that regulate nerve activity may also be occupied by LMW terpenoids. LMW terpenoids with radically diverse structural makeups influence the activity of ionotropic γ-aminobutyric acid GABA receptors, which are the targets of the organochlorine insecticides lindane and dieldrin [97]. 

The suppression of acetylcholinesterase enzyme activity in insects is another method of action for EOs, according to studies on the mechanisms of action of monoterpenoids [98]. One of the most important enzymes in the neuronal and neuromuscular connections of both insects and animals is acetylcholinesterase (AChE) [99,100,101]. AChE can be an insect-selective target for recently developed pesticides that are safe for non-target vertebrates. The insect AChE differs from the mammalian one by a single residue, known as the insect-specific cysteine residue [102,103,104,105,106]. Therefore, EOs are considered to be a possible source of pesticides due to their ability to change the AChE activity of insects [107,108,109,110,111]. The ability of EOs from the following plants to inhibit AChE has been proven: *Thymus praecox* subsp. *caucasicus*, *Cyclotrichium niveum*, *Santolina chamaecyparissus*, *Ormenis multicaulis*, *Echinacea purpurea*, *Salvia chionantha*, *Anethum graveolens*, and *Salvia lavendulaefolia* [107,108,112,113]. This activity of inhibition was evaluated for several components. Of the 73 compounds that were evaluated, 48 showed anti-AChE activity. Twenty-three of the twenty-eight substances tested on insect AChE inhibited the enzyme. The most effective were 1,8-cineole, cis-ocimene, niloticin, limonene, menthol, α-pinene, β-phellandrene, and carvacrol [110,111,113,114,115,116,117,118]. In mM concentrations, the majority of the EO components exhibited anti-AChE action. AChE in a µM concentration was shown to be inhibited by the carvacrol component of EOs in only one study [119]. According to the available data, several of the EO constituents have an inhibitory effect that is either competitive or noncompetitive [120,121,122,123,124,125]. The fact that the activity of EOs as complex compounds differs from the activity of its individual components makes it challenging to explain the method of action of EOs. For instance, whereas tea tree’s specific components are competitive inhibitors, the EO from *Melaleuca alternifolia* is an uncompetitive inhibitor. These competitive inhibitors bind to the AChE active sites and block ACh from binding. The uncompetitive inhibitors, instead, bind to different AChE sites and allosterically change how the enzyme functions. They impede the formation of the product because they bind more to the enzyme-substrate complex than to the enzyme alone. The enzyme’s maximal activity therefore declines. 

EOs act not only directly at the nervous system level, but can also influence insect behavior by repelling them. A substance that forms a vapor barrier to prevent an arthropod from touching its surface or flying to, landing on, or biting human or animal skin is referred to as a repellant. The most effective and long-lasting repellent is DEET (*N*,*N*-diethyl-m-toluamide), which has a wide range of activities. Regrettably, the use of synthetic repellents can create problems for both the environment and human health [126]. Common fumigants such as phosphine, methyl bromide, and DDVP (2,2- dichlorovinyl dimethyl phosphate) have detrimental consequences. Phosphonate is primarily to blame for suicide deaths in India. Methyl bromide has the ability to destroy the ozone layer, while DDVP is capable of causing cancer in humans. As a result, research for natural and environmentally safe repellents has increased. Currently, the repellent properties of various plants have been studied. Certain plant-based repellents are on par with or even better than synthetic repellents; however, because EOs are volatile, their efficacy is frequently transient.

Many essential-oil-producing plants, such as catnip, osage orange (hedgeapple), *Eucalyptus* spp., *Ocimum* spp., and *Cymbopogon* spp., have been thoroughly examined. Many plant oils or their constituents, such as soybean, lemon grass, cinnamon, and citronella, have been marketed as insect repellents over the past 10 years. Neem oil from *A. indica* provided complete protection from mosquitoes for 12 h when blended at 2% in coconut oil [127]. In their review of the effectiveness of EOs as insect repellents, Nerio et al. (2010) [128] discussed the effectiveness of monoterpenes (α-pinene, cineole, eugenol, limonene, terpinolene, citronellol, citronellal, camphor, and thymol) and sesquiterpenes (-caryophyllene) as well as phytol [129]. Several species of mint, clove, rosemary, thyme, eucalyptus, and others, have been found to be poisonous to a wide range of insects, including human head lice [130]. There is a long history of the use of carvones, 1,8-cineoles, and other isolates as fumigants. Although the exact method by which these oils act as fumigants is unknown, they mostly operate through the respiratory system when in the vapor phase. The high boiling point, high molecular weight, and low vapor pressure of EOs are physical characteristics that prevent their use in industrial-scale fumigation. The use of modern biotechnology can overcome this drawback. With the creation of novel insect repellent technologies, EOs might be a key component, and they might even play a greater role at specific locations in combating pest infestation. The multilevel action described so far can be traced to the complex chemical composition of the EOs. This complexity makes them particularly interesting for another reason. Due to their complex combination of components, which includes minor compounds acting synergistically, EOs are likely to be more resistant to pests that develop resistance [131]. It is particularly important to emphasize that all targets mentioned in the mechanisms of action described above are yet to be confirmed in *V. destructor*. The TRPA1 ion channel from *V. destructor* was recently described by Peng et al. (2015) [132]. They also demonstrated through their research that carvacrol and α-terpineol are two volatile chemicals that activate the TRPA1 ion channel and have a strong repelling effect on this parasitic mite. Li et al. (2017) [133] investigated the impact of *Syzygium aromaticum* EO on the enzyme activity of *V. destructor* in a different investigation.

The physiological effects of a 30 min exposure to clove EO included decreased metabolism, increased Ca^2+^Mg^2+^ATPase, glutathione-S-transferase (GST), and superoxide dismutase (SOD) bioactivities at elevated concentrations, which ultimately triggered the stress response. They also came to the conclusion that *Varroa*’s GST detoxifying ability was severely suppressed.

## 7. Application Method in Laboratory and Field Studies 

Several laboratory assays have been designed to test the acaricide efficacy of EOs on *V. destructor*. Fumigation (evaporation), total exposure (contact and fumigation), spraying, repelling, and systemic injection of the EOs were the methods used. In order to test for contact toxicity, a material (such as the bottom of a Petri dish or the inside of a glass scintillation vial) must first be treated with the test EO before the mites are added to the treated system. Fumigation tests, on the other hand, make use of two-level systems/chambers; mites usually lodge in the upper level and are separated from the lower chamber, containing an essential oil-soaked material. Other experimental assays, instead, have involved the direct spraying of EOs on mites. Finally, indirect toxicity methods were also studied that involved testing the acaricidal efficacy of oils integrated into diets fed to bees parasitized by *Varroa*. In trials where, in addition to acaricide efficacy, toxicity on bees is also being tested, mites are placed in a system where newly emerged bees are present (1–3 days). The results obtained varied widely even for the same botanical species when used independently in laboratory and field research. These discrepancies can be traced to several factors. First of all, the experimental conditions can be considered. For instance, the incubation temperatures and humidity of *Varroa* processed for toxicological analysis are parameters that have varied widely among published studies. Research groups have worked with values from a minimum of 22 °C and 60% relative humidity to a maximum of 34 °C and 70% relative humidity. Secondly, the administration technique used in each trial is mostly to blame for the widely disparate varroacidal activity results of the EOs. For instance, when the acaricide efficacy of *S. aromaticum* against *V. destructor* was evaluated, it was found that systemic treatment is a less effective delivery strategy than total exposure, with the use of complete exposure leading to substantially higher mortality rates [134]. 

EOs that have returned encouraging results in the laboratory have often been assayed in the field. Field studies were conducted by impregnating various absorbent materials with the EOs or using gas vaporizers powered by solar panels [135]. Of the various EOs examined, only a small number showed efficacy when used directly in hives, although they were evaluated and showed favorable behavior against *Varroa* mites under controlled laboratory conditions.

The importance of in vivo bioassays to verify the potency of the investigated EOs is made abundantly evident by the following finding. Differences between laboratory and field tests are the result of the interaction of many variables, including environmental conditions, the higher volatility of oils in open systems, colony strength and the ventilation of worker bees within the hive. These conditions could all affect the volatile substances used, lowering their activity. Fumigation is often the approach that proves to be the most successful in both lab and field tests. As it facilitates the molecules’ entry into the targeted organism’s respiratory system, which results in rapid knockdown and high mortality rates, this delivery approach is considered the most efficient way to administer EOs. The effectiveness and low risk to honeybees of fumigation as an administration technique for *Acantholippia seriphioides* and *Schinus molle* EOs to control *V. destructor* in a laboratory setting (16% and 8% mortality rate for honeybees, respectively, for *Acantholippia seriphioides* and *Schinus molle*), compared to the use of the complete exposure method (87% and 42% mortality rate for honeybees, respectively), has been proven [136]. The extreme pharmacological practicality of EOs when administered by fumigation techniques was also demonstrated by Bava et al. (2022) [137]. The authors found that fennel EO vapors were toxic to *Varroa*, while bees began to experience toxic effects only when subjected to doses ten times higher than those of *Varroa* [137]. Regarding this, it is also important to cite the study conducted by Hoppe (1990) [138]. Hoppe (1990) [138] tested the toxicity of 55 EOs on bees and mites. Twenty-four EOs resulted in a mite mortality of more than 90% after 72 h. Only 9 of these 24 oils resulted in bee mortality rates under 10%. Thus, special care must be made to use concentrations of these compounds that are harmful to mites yet have no or very little toxicity to bees when applying them. When used at concentrations of 5–15 g, 50–150 g, and 20–60 g per liter of air, respectively, thymol, camphor, and menthol killed almost 100% of the mites without significantly affecting the bee population [139]. Nevertheless, alterations in honeybee behavior can be seen at non-lethal dosages of EOs. For example, honeybees react differently to EO anti-varroa treatments as they get older. Older bees typically avoid Apiguard^®^ gel, although 2-day-old bees react indifferently to it [140]. Apiguard^®^ seems to turn off foragers. Apiguard^®^ contact causes strong fanning behavior to occur. The laboratory study already described by Mondet et al. (2011) indicated that forager bees exposed to Apiguard^®^ in the hive may develop a tolerance to this treatment when exposed from a young age [140]. Bergognoux et al. (2013) [141] demonstrated the effect of a topical application of the terpenoid thymol on adult honeybee’s (*Apis mellifera*) phototactic behavior. By counting the amount of time spent in the vicinity of a light source and in areas opposing it, behavior was measured under various light intensities. Positive phototaxis in the bees was induced by stimuli of 200 lx. Thymol given to bees at a rate of 1 ng/bee had no impact on their phototaxic behavior, whereas bees given 10 or 100 ng of thymol 1 h prior to the test were less attracted to the 200-lx stimulus [141]. Furthermore, thymol treatment can have negative effects at the hive level, including brood mortality and removal as well as the possibility of queen mortality, despite the fact that queens are less sensitive to thymol than workers are [142,143,144].

## 8. Analysis of Laboratory and Field Study Achievements

The Apiaceae, Asteraceae, Lamiaceae, Myrtaceae, Poaceae, and Verbenaceae plant families have been the most thoroughly investigated in research of EO activity. Tests have been conducted with both pure essential oils and isolated monoterpenes. Particular association studies have instead predicted the association of EOs with entomopathogenic fungi [145,146]. Below we mention a small number of studies, and their efficacy results, for each family that was studied. The studies exampled allow us to make some important considerations. For the family Myrtaceae, among others, the acaricidal properties of *Syzygium aromaticum* were investigated in several independent laboratory tests. *S. aromaticum* showed a wide variability in efficacy when administered as a fumigant. Sammataro et al. (1998) [147] and Vieira et al. (2012) [148] obtained similar but far superior results to Xiao- Ling et al. (2012) [149]. The first two research groups recorded an average mortality of around 87%, while the second research group recorded an average mortality of 54%. Similar non-constant acaricidal activity was recorded for the essential oils of *Mentha* spp. and *Citrus* spp. In some studies the isolated EOs returned good acaricidal efficacy, in others it was not recorded [148,150,151,152,153]. For the Apiaceae family, plants of the species *Pimpinella* spp. and *Foeniculum* spp. were evaluated in laboratory studies. While *Pimpinella* spp. vapors were found to possess an acaricidal efficacy of 92.5% in both the study of Vieira et al. (2012) [148] and Xiao-Ling et al. (2012) [149], *Foeniculum* spp. possessed a lower acaricidal capacity, often around 60% [154,155]. Few studies, instead, have concerned plants belonging to the families Verbenaceae, Lauraceae and Poaceae. For the Verbenaceae family, the species *Acantholippia seriphioides* (aerial parts) was assayed by Ruffinengo et al. in 2014 [136], which obtained a high acaricide efficacy of 99% by full exposure and 87% by fumigation. For the Lauraceae family, the species *Cinnamomum verum* [148] and *Laurus nobilis* [147] were mainly studied. Specifically, Vieira et al. (2012) [148] found an acaricidal activity by fumigation of *Cinn verum* of only 52.50% after 6 h of exposure to fumes. *Laurus nobilis*, from the same botanical family, gave better results, with an acaricidal activity close to 75% [147]. As can easily be seen, the acaricide efficacy results obtained were often different, both for oils belonging to the same family and for the same oil species, when tested in independent experiments. This discrepancy can be traced to many causes. As protocols are not standardized, comparing various works is not always simple. Simply starting from the analysis of mite sampling methods for laboratory toxicological tests, differences between the studies can be seen. In most experiments, the mites were taken directly from a brood comb by removing the wax operculum and inspecting each cell. In other experiments, massive mite recovery was achieved by anesthetizing the honeybees and the mites with carbon dioxide and then passing the sample through a sieve that allowed the mites to pass through and retained the honeybees. Finally, few studies have seen collection by powdered sugar. The former method is definitely the one that causes less traumatization of the *V. destructor* mites. The second is also a good harvesting method, as verified by Bava et al. in 2022 [137]. Powdered sugar-based methods, on the other hand, is objectionable because it subjects mites to traumatization that could affect toxicity tests. This finding is mitigated by the fact that the tests are always conducted with control groups set up with the same sampling conditions. The toxicity of the same EOs applied in different ways also differed. For example, the EO of *S. aromaticum*, in laboratory tests conducted by various groups, retained its acaricide efficacy both by contact [147,156,157] and fumigation [147], always proving harmless to bees. An independent field experimentation also yielded positive results for this EO [158]. However, in general, there were no discernible relationships between laboratory test results and field test results that would make it possible to extrapolate hive activity from laboratory data. In fact, there are situations when laboratory tests do not appear to predict activity on hives. With the exception of *C. paradisi* and *C. bergamia*, several independent studies have screened *Citrus* EOs in lab experiments without showing any promising outcomes [152,159]. Without first being vetted in laboratory tests, *C. aurantium* EO was nevertheless tested directly on hives and proved to cause an increase in dead *Varroa* and a decrease in infection rates [160]. For these reasons, it bears repeating that the absence of established protocols—including application method, time, treatment repetition, etc.—prevents result comparability between screening tests. Finally, it should be remembered that the effectiveness of different EOs varies depending on the harvest period, soil composition, sun exposure of the plant and other ecological factors. The latter turns out to be an uncontrollable factor, making it essential to investigate the phytochemical profile of EOs tested in laboratory and field tests. In Table 1 and Table 2 below several studies conducted in the last years are summarized.

## 9. Disadvantages to Overcome and Future Perspectives

To promote the commercialization and eventual use of more EO products on the market, a variety of measures are required. First and foremost, it is crucial to streamline the complicated and pricey permission process for novel botanical pesticides (BPs) based on plant extracts that have a history of usage in the food industry, the cosmetics industry, or the pharmaceutical industry. Secondarily, it is imperative to avoid losing power in the field against the targeted pests; this note emphasizes the need for powerful stabilizing techniques (e.g., encapsulation). While having good contact effects, EOs have low persistence because they quickly fumigate the environment after application and gradually their active ingredients degrade. The creation of appropriate EO formulations as active components of biopesticides (BPs) with a greater duration of activity should receive attention. The focus of this research, which is currently in its early phases, is on efficient encapsulation methods. A controlled release of oil vapors is possible thanks to existing technology, which also reduces the loss of the active substances. Encapsulation is the process by which an active component is contained or covered by a matrix wall. This matrix isolates the bioactive molecule from the environment until it is released in response to external circumstances. Although there are a variety of EO encapsulation techniques, the majority of which were developed for the food industry and for pharmaceutical applications, the use of EOs as BPs necessitates the use of low-cost encapsulating techniques. The method currently being used appears to be coacervation, commonly known as phase separation. For the use of EOs for BPs, simple coacervation, which employs a single polymer such as gelatin or ethyl cellulose, is acceptable [41]. The usage of cyclodextrins (CDs) is yet another successful tactic. To create CDs, sometimes referred to as α-, β-, and γ-CDs, six, seven, or eight glucose units are cyclically joined together. CD complexation is extensively used in foods, cosmetics, toiletries, and pharmaceutical applications. As CDs induce complex formation similar to molecular encapsulation, they can be viewed as nanoencapsulating agents. The bioactive EO molecules are isolated from one another and dispersed at the molecular level in an oligosaccharide matrix. Furthermore, sheets can be made by combining polymers and EOs. Attractive adhesive films with essential fragrances have been developed to control insects in horticulture and agriculture (Klerk’s Plastic Industries B.V., 1990) [191]. In this regard, it is encouraging that a significant number of commercial products that contain EOs for use in food and beverage preparation have been fully authorized by the Food and Drug Administration (FDA) and Environmental Protection Agency (EPA) in the United States as “Generally Recognized as Safe” (GRAS) items [192].

Another difficulty is that many potential EOs with effective activity originate from plants whose cultivation is expensive or undesirable due to low EO yields. Even commercially developed EO-generating plants can be challenging to care for. Moreover, monoterpene concentrations vary according to the phenological stage of the plant and are influenced by temperature and circadian rhythm [153]. Finally, the secondary metabolism of the plant and the composition of EO are directly impacted by soil acidity and climate (heat, photoperiod, and humidity). Thus, it can be difficult to obtain a standardized product, which is crucial for regulatory and marketing reasons. In an effort to enhance EO production and standardize their qualitative and quantitative qualities, elicitation products, genetic engineering, and new plant-growing technologies have all been suggested as answers to this issue. Research must also be conducted on innovative methods for extracting EOs from plants. To isolate EOs from plants today, traditional/conventional methods are employed (i.e., by standard distillation of the plant material). Throughout the past few decades, spending on new technologies (such microwaves and ultrasound) has led to the creation of efficient extraction techniques (i.e., reduction in extraction time and energy consumption, increase in extraction yield, improvement in EO quality).

Other shortcomings that can be deduced from the analysis of the literature are related to field studies. The majority of the published research on the biological efficacy of EOs focuses on evaluating the effectiveness of various EOs against various target organisms. As a result, the majority of research is still in too-early stages for developing innovative botanical pesticides (BPs). Even though this research is essential for the development and approval of BPs, there have not been many studies that have looked at how EOs harm non-target organisms. When seeking *V. destructor* selectivity, it is also important to take into account harmlessness towards the non-target. Finally, the ability of the oils to act under the wax operculum should be evaluated. The ability of the tested products to enter brood cells and hence inhibit the mite reproductive stages influences the mode of administration. If this quality is inadequate, it must be decided whether treatments need to be repeated while taking into consideration the lag time needed for the subsequent generation of *V. destructor* to appear. In relation to this, the application methods must also be considered, with an emphasis on the supports and volatilization rates, since these variables affected the amount of EO released inside the hives. Volatilization rates are affected by the support, internal hive airflow, and temperature variations [193]. Depending on the support, EOs have been shown to release differently [194,195,196]. The importance of carefully outlining application processes is best illustrated by the EO of *Acantholippia seriphioides*. The activity discovered, albeit good, is not selective (selectivity ratio as LD50-bees/LD50-*Varroa* = 1:3) when *A. seriphioides* EO is applied allowing for entire exposure (vapour and direct contact). In contrast, selectivity was obtained when the same EO was micro-encapsulated (gum Arabic) and applied by vaporization (1:30% *Varroa*/bees mortality after 72 h exposure). This pattern of maintaining insecticidal activity when switching from contact to fumigation is not typical, though. For instance, it was discovered that geraniol has high contact toxicity but minimal fumigant activity against *Tribolium castaneum* [159]. On the other hand, when the application mode was changed, the EOs of thyme, spearmint, savory, and dillum retained the same varroacidal activity [159]. 

## 10. Residues 

For mite management, EOs and their constituent parts have been extensively assayed with various degrees of effectiveness. However, there is not much research into residues in honey and other hive products. Because EOs are complex chemical compounds and because most honeys naturally contain many EOs components, residue analysis following treatment can be difficult and inconclusive. As a result, compliance with EU and US federal rules may be challenging. Due to their ability to affect the taste and the quality of the honey as well as pose health problems, residues are a significant concern. Currently authorized products for the control of *V. destructor* are mainly thyme-based. According to EU Regulation No. 2377/90, Thymol belongs to Category II of non-toxic veterinary drugs, which do not need an MRL (maximal residue limit). However, because they have a strong scent, pharmaceutical preparation based on EOs can change the flavor of honey even when used in very little amounts. Bogdanov et al. (1999) [197] described the results of a sensory analysis performed by a panel of experts. The results determined that thymol at concentrations of 1.1-1.3 mg/kg affected the flavor of honey. The threshold concentration for altering the organoleptic properties of honey was highest for camphor (5–10 mg/kg) and menthol (20-30 mg/kg). The contaminated products had an astringent and “medicinal” flavor, according to the participants [197]. 

## 11. Conclusions

The use of EOs in beekeeping is currently a fascinating field of research. EOs must typically be synthesized as a microemulsion or nanoemulsion due to their physicochemical limitations, specifically their volatility and low bioavailability of active polyphenolic components. Future studies might concentrate on employing commercially available surfactants to apply aqueous microemulsions. The usage of natural surfactants might provide another element of “greenness”. The development of novel formulations using polymer-based nanocapsules or encapsulation with metal nanoparticles using nanotechnology may also boost the availability of EOs, while also enhancing their functions. The scientific community’s efforts to standardize laboratory and field methods should be a key factor in future investigations. Comparable outcomes for the investigated botanical species might result from standardizing laboratory procedures. Furthermore, since field studies are less consistent than laboratory studies, more investigation in this area is needed to close knowledge gaps and validate findings obtained in challenging environmental settings.

## Figures and Tables

**Table 1:** Overview of significant laboratory experiments on EOs conducted for *V. destructor*.

Family	Botanical Name	*Varroa destructor* Toxicity	Honeybee Toxicity	Method of Administration	Reference (Ordered by Year of Publication)
Myrtaceae	*Syzyygium* spp.	Mortality rate > 80% at 1% concentration	Equal to untreated control group	Complete exposure	Kraus et al. (1990) [161]
Lamiaceae	*Origanum* spp.	Mortality rate 100% at 10% concentration	Mortality rate 20% at 10% concentration	Complete exposure	Kraus et al. (1990) [161]
Myrtaceae	*Syzyygium aromaticum*	Mortality rate of 87.2%	Not evaluated	Fumigation	Sammataro et al. (1998) [147]
Myrtaceae	*Melaleuca alternifolia*	Mortality rate of 59.4%	Not evaluated	Fumigation	Sammataro et al. (1998) [147]
Lauraceae	*Laurus nobilis*	Mortality rate of 75.5%	Not evaluated	Fumigation	Sammataro et al. (1998) [147]
Urticaceae	*Urtica dioica*	Mortality rate of 25–80%	Non toxic	Fumigation	Ruiz et al. (1998) [162]
Rutaceae	*Ruta graveolens*	Mortality rate of 100%	100% mortality rate	Fumigation	Ruiz et al. (1998) [162]
Myrtaceae	*Syzygium aromaticum*	100% at best dose (1 mg) and best time (after 48 h)	*Apis mellifera* LD50 estimates were not available for clove oil because of low bee mortality at all doses assayed	Complete exposure	Lindberg et al. (2000) [156]
Lamiaceae	(1) *Satureja hortensis* (2) *Salvia rosmarinus* (3) *Lavandula angustifolia* (4) *Origanum majorana* (5) *Mentha spicata*	All the essences caused more than 97% mortality at 2% of concentration	Bee mortality ranged from 2–3% for thyme, spearmint, lavender and savory; Marjoram, rosemary caused 4–14% bee mortality	Contact in Petri dish	Ariana et al. (2002) [159]
Asteraceae	*Tagetes minuta*	LD50 = 4.37 mg after 24 h	At the highest concentration (5%), the oil did not exhibit bee toxicity.	Complete exposure	Eguaras et al. (2005) [163]
Asteraceae	(1) *Eupatorium buniifolium* (2) *Tagetes minuta* (3) *Wedelia glauca*	LD50 = 5.1077 LD50 = 3.2209 LD50 = 0.5903	LD50 = 7.7885 LD50 = 12.3068 LD50 = 1.0925	Complete exposure in Petri dish	Ruffinengo et al. (2005) [164]
Anacardiacae	*Schinus molle*	LD50 = 1.3302	LD50 = 23.5647	Complete exposure in Petri dish	Ruffinengo et al. (2005) [164]
Verbenaceae	(1) *Aloysia polystachya* (2) *Acantholippia seriphioides* (3) *Lippia turbinata* (4) *Lippia junelliana*	LD50 = 4.9819 LD50 = 1.0980 LD50 = 2.2290 LD50 = 1.9847	LD50 = >25 LD50 = 1.2217 LD50 = 3.9751 LD50 = 4.0749	Complete exposure in Petri dish	Ruffinengo et al. (2005) [164]
Lamiaceae	*Minthostachys mollis*	LD50 = 6.6027	LD50 = 11.7725	Complete exposure in Petri dish	Ruffinengo et al. (2005) [164]
Asteraceae	*Heterothalamus alienus*	LC50 = 1.37 mg/cage after 48 h	LC50 = 5.51 mg/cage after 48 h	Complete exposure in Petri dish	Ruffinengo et al. (2006) [165]
Rutaceae	(1) *Citrus paradisi* (2) *Citrus sinensis*	(1) 76% mortality at 8 µL/Petri dish (2) 40% mortality at 40 µL/Petri dish	Not observed	Contact in Petri dish	Fuselli et al. (2009) [152]
Lamiaceae	(1) *Lavandula officinalis* (2) *Lavandula hibrida* (3) *Thymus vulgaris*	(1) LD50 = 2.24 after 72 h (2) LD50 = 7.95 after 72 h (3) LD50 = 2.93 after 72 h	(1) LD50 = >20 after 72 h (2) LD50 = >20 after 72 h (3) LD50 = 8.05 after 72 h	Complete exposure in Petri dish	Ruffinengo et al. (2009) [166]
Lamiaceae	(1) *Origanum vulgare* (2) *Mentha spicata*	(1) LC50 = 56.1 µg/vial after 4 h (2) LC50 = 173.2 µg/vial after 4 h	(1) LC50 = 331.3 µg/bee after 4 h (2) LC50 = 523.5 µg/bee after 4 h	Contact in glass scintillation vials	Gashout and Guzmán-Novoa (2009) [157]
Myrtaceae	*Eucalyptus globulus*	LC50 (μL Petri dish^−1^) = 11.7 after 72 h	LC50 (μL Petri dish^−1^) = >20 after 72 h	Complete exposure in Petri dish	Gende et al. (2010) [167]
Lamiaceae	(1) *Salvia rosmarinus* (leaves air dried) (2) *Salvia rosmarinus* (leaves oven dried)	(1) LC50 (µL per Petri dish) = >20 after 72 h (2) LC50 (µL per Petri dish) = 7.07 after 72 h	(1) LC50 (µL per Petri dish) = >20 after 72 h (2) LC50 (µL per Petri dish) = >20 after 72 h	Complete exposure in Petri dish	Maggi et al. (2010) [168]
Myrtaceae	*Syzygium aromaticum* (floral buds)	LC50 = 0.59 µL/dish after 24 h	LC50 = 15.53 µL/dish after 24 h	Complete exposure	Damiani et al. (2011) [169]
Asteraceae	*Baccharis flabellate*	LC50 = 1.14 after 48 h	LC50 = >10 after 48 h	Spraying application in Petri dish	Damiani et al. (2011) [169]
Asteraceae	(1) *Tagetes minuta* (leaves of bloomed plant) (2) *Tagetes minuta* (leaves of not-bloomed plant) (3)*Tagetes minuta* (flowers)	(1) 97.7% after 6 h (2) 98.3% after 6 h (3) 100% after 6 h	24.4% after 6 h	Contact in Petri dish	Chamorro et al. (2011) [170]
Lamiaceae	*Thymus kotschyanus* (leaves)	LC50 = 1.07 µL/L air	LC50 = 5.08 µL/L air	Fumigation in Petri dish	Ghasemi et al. (2011) [171]
Myrtaceae	*Eucalyptus camaldulensis*	LC50 = 1.74 µL/L air	LC50 = 3.05 µL/L air	Fumigation in Petri dish	Ghasemi et al. (2011) [171]
Lamiaceae	*Minthostachys verticillata*	LC50 = 1.44 after 48 h	LC50 = >10 after 48 h	Spraying application in Petri dish	Damiani et al. (2011) [169]
Apiaceae	*Pimpinella asinum*	92.5% after 6 h at 200 µL	3.7% after 6 h at 200 µL	Fumigation	Vieira et al. (2012) [148]
Lamiaceae	*Salvia rosmarinus*	77.5% after 6 h at 200 µL	3.7% after 6 h at 200 µL	Fumigation	Vieira et al. (2012) [148]
Lamiaceae	*Mentha* spp.	47.5% after 6 h at 200 µL	6.2% after 6 h at 200 µL	Fumigation	Vieira et al. (2012) [148]
Lauraceae	*Cinnamomum verum*	52.5% after 6 h at 200 µL	5% after 6% at 200 µL	Fumigation	Vieira et al. (2012) [148]
Myrtaceae	*Syzygium aromaticum*	87.5% after 6 h at 200 µL	13.75% after 6% at 200 µL	Fumigation	Vieira et al. (2012) [148]
Apiaceae	*Pimpinella asinum*	92.5% after 48 h	Not registered	Fumigation	Xiao-ling et al. (2012) [149]
Myrtaceae	*Syzygium aromaticum*	54% after 48 h	Not registered	Fumigation	Xiao-ling et al. (2012) [149]
Asteraceae	*Eupatorium buniifolium* (leaves)	80% after 48 h	13% after 48 h	Fumigation	Umpiérrez et al. (2013) [172]
Verbenaceae	*Acantholippia seriphi-oides* (microencapsulated oil)	99% after 72 h	54% after 72 h	Complete exposure in Petri dish	Ruffinengo et al. (2014) [136]
Anacardiacae	*Schinus molle* (microencapsulated oil)	87% after 72 h	42% after 72 h	Complete exposure in Petri dish	Ruffinengo et al. (2014) [136]
Lamiaceae	(1) *Thymus kotschyanus* (aerial parts) (2) *Mentha longifolia* (aerial parts)	(1) 84.4% after 10 h; (2) 65.5% after 10 h	(1) 7.2% after 10 h; (2) 10.13 after 10 h	Fumigation	Ghasemi et al. (2016) [150]
Myrtaceae	*Eucalyptus camaldulensis* (aerial parts)	71 % after 10 h	12% after 10 h	Fumigation	Ghasemi et al. (2016) [150]
Apiaeceae	*Ferula gummosa* roots	49.9% after 10 h	26% after 10 h	Fumigation	Ghasemi et al. (2016) [150]
Poaceae	*Cymbopogon* *citratus*	LC50 = 474.13 µg/mL after 4 h	LD50 = 54,844.0 µg/mL after 4 h	Contact in glass scintillation vials	Sabahi et al. (2018) [173]
Asteraceae	*Tagetes lucida*	LC50 = 1256.27 µg/mL after 4 h	LD50 = 83,297.0 µg/mL after 4 h	Contact in glass scintillation vials	Sabahi et al. (2018) [173]
Apiaceae	*Foeniculum vulgare*	LD50 (µL) = 1.837 after 48 h	LD50 (µL) = 4.055	Fumigant toxicity in two level cage	Lin et al. (2019) [154]
Leguminosae	*Dalbergia odorifera*	LD50 (µL) = 12.212 after 48 h	LD50 (µL) = 24.646 after 48 h	Fumigant toxicity in two level cage	Lin et al. (2019) [154]
Lamiaceae	(1) *Mentha haplocalyx* (2) *Pogostemon* spp.	(1) LD50 (µL) = 2.274 after 48 h (2) LD50 (µL) = 2.047 after 48 h	LD50 (µL) = 5.003 after 48 h 2) LD50 (µL) = 3.745 after 48 h	Fumigant toxicity in two level cage	Lin et al. (2019) [154]
Zigiberaceae	*Amomum tsao-ko*	LD50 (µL) = 2.548 after 48 h	LD50 (µL) = 3.769 after 48 h	Fumigant toxicity in two level cage	Lin et al. (2019) [154]
Cannubaceae	*Humulus lupulus* (flowers) *victoria* variety	LC50 (μL/mL) = 2.7 after 48 h	NOAEL of 5 μL/mL (X2(1, N = 50) = 5.35, *p* = 0.02)	Complete exposure in Petri dish	Iglesisas et al. (2020) [174]
Rutaceae	(1) *Citrus paradisi* (2) *Citrus limon* (3) *Citrus bergamia* (4) *Citrus sinensis* (5) *Citrus reticulata*	(1) 65% after 1 h (2) 82% after 1 h (3) 77% after 1 h (4) 89% after 1 h (5) 67% after 1 h	No mortality was reported	Contact in Eppendorf tube	Bava et al. (2021) [153]
Chenopodiaceae	*Chenopodium ambrosioides*	LD50 = 5.238 mL/Lair	Not evaluated	Fumigation in glass vial	Aglagane et al. (2022) [151]
Lamiaceae	*Mentha suaveolens* subsp. *timija*	LD50 = 3.360 µL/Lair	Not evaluated	Fumigation in glass vial	Aglagane et al. (2022) [151]
Lauraceae	*Laurus nobilis*	LD50 = 5.470 µL/Lair	Not evaluated	Fumigation in glass vial	Aglagane et al. (2022) [151]
Lamiaceae	*Melissa officinalis*	100% (concentration 25 µL/L air) after 25 h	1.7% after 25 h	Fumigation in two level cage	Karimi et al. (2022) [175]
Fagaceae	*Quercus infectoria*	100% (concentration 25 µL/L air) after 25 h	1.7% after 25 h	Fumigation in two level cage	Karimi et al. (2022) [175]
Caesalpiniaceae	*Ceratonia siliqua*	100% (concentration 25 µL/L air) after 25 h	2% after 25 h	Fumigation in two level cage	Karimi et al. (2022) [175]
Lamiaceae	*Origanum heracleoticum*	90.9% at a concentration of 2 mg/mL (contact); 84% after 90 min. (fumigation)	No mortality was reported	Contact toxicity in Eppendorf tube and fumigation in Eppendorf tube	Castagna et al. (2022) [176]
Apiaceae	*Foeniculum vulgare* sbps. *piperitum* (whole plant)	68% in Eppendorf tube, after 48 h, and at concentration of 2 mg/mL; 53.3% in two level cage, after 48 h, and at concentration of 40 mg/mL	At a concentration of 7% (70 mg/mL), after 48 h, 80% of the tested honeybees died	Fumigation in Eppenderf tube and in two level cage	Bava et al. (2022) [137]

**Table 2:** Overview of significant field experiments on EOs conducted for *V. destructor*.

Family	Botanical Name	*Varroa destructor* Toxicity	Honeybee Toxicity	Method of Administration	Reference (Ordered by Year of Publication)
Lamiaceae	Water emulsion of *Thymus* spp. (1%) and *Salvia* spp. (0.5%)	95% mortality rate	Not evaluated	Aerosol treatment repeated four times at intervals of 3–4 days	Colin et al. (1990) [177]
Lamiaceae	(1) *Lavandula coronopifolia* (2) *Menta piperita*	(1) No effect (2) No effect	(1) Not evaluated (2) Not evaluated	Fumigation	Al-Abbadi and Nazer (2003) [178]
Myrtaceae	*Eucalyptus* sp.	50% mortality rate	Not evaluated	Fumigation	Principal et al. (2005) [179]
Rutaceae	*Citrus aurantium*	General reduction in infestation rate	Brood rearing activity increased	Fumigation	Abd El-Wahab and Ebada (2006) [160]
Poaceae	*Cymbopogon winteranius*	General reduction in infestation rate	Brood rearing activity increased	Fumigation	Abd El-Wahab and Ebada (2006) [160]
Poaceae	*Cymbopogon flexuosus*	General reduction in infestation rate	Brood rearing activity increased	Fumigation	Abd El-Wahab and Ebada (2006) [160]
Rutaceae	*Ruta graveolens*	83% after 24 h	Not evaluated	Fumigation	Castagnino and Orsi (2012) [180]
Myrtaceae	*Eucalyptus* spp.	86.4% after 24 h	Not evaluated	Fumigation	Castagnino and Orsi (2012) [180]
Lamiaceae	*Mentha piperita*	81.3% after 24 h	Not evaluated	Fumigation	Castagnino and Orsi (2012) [180]
Lamiaceae	*Lavandula officinalis* (leaves)	Average mortality calculated at 3 years = 78.9%	Not evaluated	Fumigation	Kütükoğlu et al. (2012) [181]
Apiaceae	*Foeniculum vulgare* (leaves)	Average mortality calculated at 3 years = 70.5%	Not evaluated	Fumigation	Kütükoğlu et al. (2012) [181]
Lauraceae	*Laurus nobilis* (leaves)	Average mortality calculated at 3 years = 70.9%	Not evaluated	Fumigation	Kütükoğlu et al. (2012) [181]
Amaryllidaceae	*Allium sativum*	76.7% average mortality	Not evaluated	Strip of blotting paper soaked with 5 mL of EO	Goswami and Khan (2013) [182]
Lauraceae	*Cinnamomum verum*	80.9% average mortality with a mixture of EO (30%), olive oil (70%) and talcum powder	Not evaluated	Fumigation	El-Hady et al. (2015) [183]
Apiaceae	*Pimpinella anisum*	80% average mortality with a mixture of EO (30%), olive oil (70%) and talcum powder	Not evaluated	Fumigation	El-Hady et al. (2015) [183]
Verbenaceae	*Lippia berlandieri*	74% mite mortality obtained with 1.16 mL of EO after 21 days	Not evaluated	Fumigation	Romo- Chacón et al. (2016) [145]
Lamiaceae	*Thymus algeriensis*	32.6% mortality after two months treatment	No negative effect on the brood	Spraying	Kouache et al. (2017) [184]
Lamiaceae	*Origanum elongatum* (foliar biomass)	81.8% after one day of treatment	Not observed	Fumigation	Ramzi et al. (2017) [185]
Lamiaceae	*Thymus satureioides* (foliar biomass)	60.8% after one day of treatment	Not observed	Fumigation	Ramzi et al. (2017) [185]
Lamiaceae	Blend of *Thymus satureioides* and *Origanum elongatum*	93.9% after one day of treatment	Not observed	Fumigation	Ramzi et al. (2017) [185]
Lamiaceae	*Origanum vulgare*	97.4% after 4 weeks	Equal to untreated control group	Electric vaporizer with 20 mL of oregano oil	Sabahi et al. (2017) [135]
Lamiaceae and Myrtaceae	Blend of *Origanum vulgare* and *Syzygium aromaticum*	57.8% after 4 weeks	Not evaluated	Fumigation	Sabahi et al. (2017) [135]
Fabaceae, Ginkgoaceae, Febaceae and Lamiaceae	Blend of *Sophora flavescens*, *Ginkgo biloba*, *Gleditsia sinensis* and *Teucrium chamaedrys*	80.8% after 20 days	Not evaluated	Fumigation	Stanimirović et al. (2017) [186]
Myrtaceae	*Azadirachta indica*	82.6% after 72 h	Not evaluated	Fumigation	Bakar et al. (2017) [187]
Myrtaceae	*Eucalyptus globulus* (leaves)	15.6% using 1 mL/week for 3 weeks	Not evaluated	Fumigation	Merabet et al. (2018) [188]
Myrtaceae	*Eucalyptus globulus* (leaves) and *thymol*	57% using 1 mL/week for 3 weeks	Not evaluated	Fumigation	Merabet et al. (2018) [188]
Lamiaceae	*Salvia officinalis* (aerial parts)	Calculated infestation rate before treatment 16.24%; infestation rate after treatment 0.9% with a dose of 20%	Not evaluated	Fumigation	Bendifallah et al. (2018) [189]
Myrtaceae	*Eucalyptus amygdalina* (leaves)	Mean mortality 14.1%	Not evaluated	Fumigation	A. Merabet et al. (2020) [190]

## Data Availability

Not applicable.
